# High-frequency Coastal Overwash Deposits from Phra Thong Island, Thailand

**DOI:** 10.1038/srep43742

**Published:** 2017-03-09

**Authors:** Chris Gouramanis, Adam D. Switzer, Kruawun Jankaew, Charles S. Bristow, Dat T. Pham, Sorvigenaleon R. Ildefonso

**Affiliations:** 1 Department of Geography, National University of Singapore, 117570, Singapore; 2 Earth Observatory of Singapore Nanyang Technological University Singapore, 639798, Singapore; 3 Asian School of the Environment Nanyang Technological University Singapore, 639798, Singapore; 4 Department of Geology, Faculty of Science, Chulalongkorn University Bangkok, 10330, Thailand; 5 Department of Earth and Planetary Sciences Birkbeck College, University of London London, WC1E 7HX, United Kingdom; 6 Viet Nam Institute of Meteorology, Hydrology and Climate Change, Hanoi, Vietnam

## Abstract

The 26^th^ December 2004 Indian Ocean Tsunami (IOT) emanated from an Mw 9.2 earthquake that generated a 1600 km-long rupture along the Sumatran Megathrust and generated tsunami waves up to 30 m high. The IOT directly impacted the Bay of Bengal and east Africa, with over 283,000 people perishing. At the time, this catastrophic event was considered unprecedented and sparked intense investigations to test this claim. It is now believed that four pre-2004 IOT events have occurred in the last 2500 years, recurring every 550 to 700 years. Much of this information comes from Phra Thong Island, Thailand, where a sequence of four stacked sandsheets separated by organic units has been recognised and compared to the 2004 IOT event. Recently, ground-penetrating radar on Phra Thong Island identified a region that could not be explained by the known stratigraphy. The stratigraphy of the area was investigated from auger cores and pits, and several previously-unrecognised sandsheets were identified and compared to the known tsunami sandsheets. The proximity of the newly-recognised sandsheets to the palaeo-coastline of Phra Thong Island does not preclude the impacts of localised storms in sandsheet emplacement or that tsunamigenic earthquake recurrence may have been more frequent in the past.

The devastating trans-oceanic 2004 Indian Ocean Tsunami (IOT)^1^,^2^,^3^ has sparked a large amount of research in the countries impacted by the IOT^4^,^5^,^6^,^7^,^8^,^9^, as this catastrophic event was historically unprecedented in this region^10^,^11^,^12^,^13^,^14^,^15^,^16^,^17^,^18^. Two key aspects of this research drive has been to characterise sedimentary signatures of tsunami in coastal environments^4^,^19^,^20^ and to identify earlier tsunami deposits to quantify the recurrence intervals for these events^21^,^22^. The latter is of paramount importance in predicting future events and mitigating against future catastrophes^23^,^24^,^25^.

One of the key sites in determining the palaeo-tsunami history emanating from the Sumatran Megathrust is the northern beach ridge plain of Phra Thong Island, Thailand (Fig. 1a)^21^. Here, the 2004 IOT wave height has been variably measured at 6.6 m^26^ and 20 m^21^.

The geomorphic history of Phra Thong Island, based on quartz optically stimulated luminescence (OSL) dating of the beach ridge sequence, shows that the eastern portion of the island contains beach ridges formed during the last interglacial period^27^. The western portion also contains beach ridges that have variably prograded westwards and been sequentially eroded from the mid-Holocene^28^. Interspersed between the younger beach ridge sequence are a series of swales that preserve sand sheets ascribed to tsunami deposits^21^. The 2004 IOT (Sandsheet A) and three earlier sand sheets (Sandsheets B, C and D) have been recognised from two adjacent swales located approximately 400 metres (Swale X) and 550 metres (Swale Y) east of the modern, western coastline of Phra Thong Island (Fig. 1b–d)^21^. Two palaeo-sand layers beneath the 2004 IOT layer were recognised along a transect north of this location (Fig. 1c)^29^. Another sand sheet (Sandsheet X) was recognised between Sandsheet’s A and B from a site located in Swale VI approximately 1700 metres northeast of Swale Y and 1400 metres east of the modern coastline (Fig. 1c)^30^.

On Phra Thong Island, the 2004 IOT is recognised as a landward-fining, medium- to very-fine sand that varies in with little sediment deposited on the beach ridges but thicker deposits in the swales^21^,^31^. The 2004 IOT deposit has a sharp basal contact, commonly occurring as a horizontal bed that fines upward from medium sand to coarse silt and occasionally coarsens upward from medium to silty fine sand^20^,^21^. Within the 2004 IOT deposit are seaward dipping cross beds and laminae with mud drapes, parallel silty laminae, muddy rip-up clasts, leaves, abundant marine, beach and subtidal diatom species and few freshwater diatom species, and bivalve shell fragments^20^,^21^,^30^,^32^,^33^,^34^. The 2004 IOT sediments have a bimodal sediment distribution that is dominated by quartz (85 to 95%), benthic foraminifera and shell fragments (8 to 15%), muscovite (4%), heavy minerals (1.7 to 3%) and a low organic content (1.5 to 2.5%)^30^,^32^. The <0.125 mm component as determined by X-ray diffraction (XRD) is dominated by quartz (52 to 57%), but feldspars (orthoclase and microcline), aragonite, zircon, muscovite, monazite, kaolinite, cassiterite and labradorite are present, attesting to the nearshore marine sediment source^35^.

The four older sand sheets preserved in Swales X and Y show strong sedimentological similarities (sharp erosional contacts, fining upward unimodal coarse sand to coarse silt, containing leaf or bark fragments) to the 2004 IOT deposit, but lack many of the sedimentary structures, microfauna and microflora^20^,^21^,^30^. The palaeo-sand sheets from Swale Y lack preserved diatoms and carbonate microfauna (foraminifera), likely due to temperature-affected dissolution^20^,^21^,^30^,^32^, although it is unclear why the same processes would not have affected the carbonate and unabraided shell material in the intertidal deposits preserved at the base of the swale^21^,^32^. Sandsheets B and C were correlated between Swales X and Y, but Sandsheet D was recognised in Swale Y only^21^,^36^. Three of the four palaeo-sand sheets from Swale VI also correlate with Sandsheets B, C and D in Swale Y^30^, reinforcing the spatial extent of the palaeo-sand sheet coverage across the northern beach ridge system on Phra Thong Island^21^.

The buried sand sheets were postulated to have been deposited following tsunami events like the 2004 IOT, which is supported by the similarity of the sedimentology of the older deposits. Furthermore, the trajectory of historical storms that cross the Thai-Malay Peninsular are incapable of generating the storm surge set-up to comprehensively inundate the island^21^. Between 1945 and 1996 nine storms passed within 180 nautical miles of Phuket, none of which generated sufficient set-up to inundate the west coast of Thailand^37^. However, on the 1^st^ May 2007 a tropical depression (TD2) formed in the northern Gulf of Thailand, crossed the Malay-Thailand Peninsular and moved into the Andaman Sea on the 2^nd^ May^38^. This storm produced sufficient set-up to overtop the youngest berm (*ca*. 1.3 m above mean sea level, masl) and deposit two small (~30 × 50 m and ~20 × 15 m) overwash fans (Fig. 1d). The smaller fan is located 490 m northwest of Swale Y and the sediments were deposited on top and behind the 2007 berm. The larger fan occurs 380 m west of Swale Y within the partially recovered 2004 IOT backwash scour. The poorly formed berm in front of the scour was easily breached by the storm and the accommodation space afforded by the recovering tsunami scour allowed further inland penetration and thick storm deposits to form (Fig. 1d). This implies that storms can deposit sediment on Phra Thong Island, but from this solitary example, fan development may be restricted to a few 10 s of metres from the modern shoreline – unlike the kilometre-scale inundation and sedimentation caused by the 2004 IOT. The granulometric properties of the sediments collected from the washover fans are statistically indistinguishable from the modern-day beachface sediments and are geochemically similar to Sandsheets A and C, nearshore and beachface deposits suggesting a local and similar source of sediments supplying Sandsheets A and C and the storm deposit^35^.

To determine the recurrence interval of tsunami that have inundated Phra Thong Island and deposited the sand sheets, an intensive dating campaign has been undertaken^21^,^29^,^36^,^39^. In Swale Y, radiocarbon dates of bark, plant fragments and shells from the organic muds above and below the sand sheets were examined to obtain event bounding ages, and leaves collected from within the sand sheets to date the deposits^21^. Bulk radiocarbon dates on the organic muds above and below the sand sheets were also collected^29^. Quartz OSL dates from the Swale X and Y sand sheets and surrounding beach ridges were collected to constrain the timing of the development of the swales and deposition of the sand sheets in Swale Y^36^ and Swale VI^30^. These findings all suggest that deposition of the palaeo-sand sheets on Phra Thong Island occurred from inundation events similar to the 2004 IOT event at approximately 550 to 700 year intervals^30^,^36^. This recurrence interval has been approximated from other studies of sand sheets and archaeological records recovered from beach ridge archives elsewhere from western Thailand^40^, Sumatra^22^,^41^,^42^ and India^6^,^7^. Prior to 3800 cal. yr. BP, the recurrence interval determined from palaeotsunami sandsheets from near Aceh spans *ca*. 600 to 900 years^43^. However, precisely-dated (U/Th) coral microatolls from Simuelue Island, Indonesia suggest that two similar magnitude great earthquakes occurred within approximately 50 years at *ca*. 1390 and 1455CE^17^.

Here we present results from new auger cores and pits collected from the south-western section of Swale Y (Fig. 1), where high-frequency ground-penetrating radar^44^ identified a complex stratigraphy with more sand sheets than had been previously recognised from this swale. The new sand sheets may confound the consensus model presented above for palaeotsunami deposition on Phra Thong Island and have implications for Sunda Trench 2004 IOT-type tsunami recurrence^21^,^30^,^36^.

## Results

The south-western section of Swale Y preserves at least three more sand sheets than has been described from elsewhere in Swale Y^21^ and at least two more sand sheets than have been identified from Swale VI^30^. The stratigraphy recovered from Auger 10 and Pit 2 (Fig. 2) are used to investigate the stratigraphy of the south-western section of Swale Y. The 89 cm of sediment recovered from Auger 10 penetrated through to the beach ridge sands underlying the swale sediments at 84 cm, and thus, there is no deeper sedimentary record of tsunami sediments within the swale (Fig. 2a). Pit 2, located less than 30 cm west of Auger 10, did not extend to the total depth of the swale due to the elevated groundwater table and only the upper 78.5 cm of the stratigraphy was examined here (Fig. 2b).

Much of the stratigraphy of Pit 2 and Auger 10 mirror the stratigraphy reported elsewhere from Swale Y (Fig. 3^21^; Supplementary Information). Here we present the sedimentology of the new sand sheets and their stratigraphic placement recovered from Auger 10 (Fig. 2).

Two new sand sheets occur within the lowest stratigraphic unit above the base of Swale Y. This lowest unit, extending from 84 cm to 46 cm (Auger 10) and 45 cm (Pit 2) is a clastic medium silty to very coarse silty fine sand, with small black charcoal or diagenetically altered plant material. This unit has been interpreted as an intertidal sand elsewhere in Swale Y^21^. Within this intertidal unit in Auger 10, two thin, light grey, clean, quartz-rich, fine to medium sand layers occur from 80.5 to 80 cm and 77 to 75 cm depth. The lower contact of each layer is sharp, but the upper contact grades into the overlying sediments and no sedimentary structures are observed. Following from the nomenclature established previously^21^, these layers are named Sandsheets F and E, respectively.

Between 51 and 50 cm in Auger 10, is a dipping, gun-metal blue, quartz-rich, silty fine sand with sharp upper and lower contacts. This sand sheet is found in other auger cores collected from Swale Y^44^ (Fig. 3). The stratigraphic positioning, wholly contained within the intertidal sands, and distinctive colouration suggests that this sand sheet is a correlative of Sandsheet D found in Swale Y^21^.

Sandsheets A, B and C recovered from Pit 2 and Auger 10 are identical to the correlative sandsheets elsewhere on Phra Thong Island^21^,^29^,^30^.

Auger 10 also records a further clear, tan fine sand sheet between 19.5 and 19 cm, and which has no correlative elsewhere in Swale Y^21^. This sand sheet is distinct from the stratigraphically higher 2004 IOT Sandsheet A (base at 18 cm in Auger 10) by a 1 cm thick, dark brown, organic-rich (11 to 25% organics), muddy sand to sandy mud, with a high content of plant stems, leaves and rootlets. This unit signifies a return to typical swale-like organic mud accumulation^21^. The sand sheet is stratigraphically identical to Sandsheet X from Swale VI^30^.

## Discussion

The sedimentological and stratigraphic analysis of new sediment archives from Swale Y on Phra Thong Island^21^ raises several questions about the identification of the causal mechanism of sand emplacement and the recurrence interval of sand sheets preserved at this site.

From closely spaced auger cores it is possible to trace the sand sheets, variations in thickness of each unit and variations in the stratigraphic placement of each sand sheet. Sandsheets A and C extend across the swale (Fig. 3)^21^. Sandsheets B and D are also found in Swale Y but have a patchy distribution (Fig. 3)^44^,^45^. Direct dating of the sand sheets using quartz OSL^30^,^32^,^36^,^39^, and bounding ages of the sandsheets using ^14^C dating of bulk sediments^29^, plant and leaf fragments and buried shells^21^ have helped constrain the recurrence interval of sand sheet deposition on Phra Thong Island. Sandsheets B to D were likely formed during tsunami inundation following great earthquakes from the Sunda Trench subduction zone, of which Sandsheet A is a modern analogue^21^. However, the discovery of a sand sheet immediately between the Sandsheets A and B^30^ (Sandsheet X) and additional sand sheets found between Sandsheet D and the base of Swale Y in Auger cores 8, 9 and 10^44^ (Fig. 3), imply a more complex overwash history than has thus far been presented.

**Figure 1:**
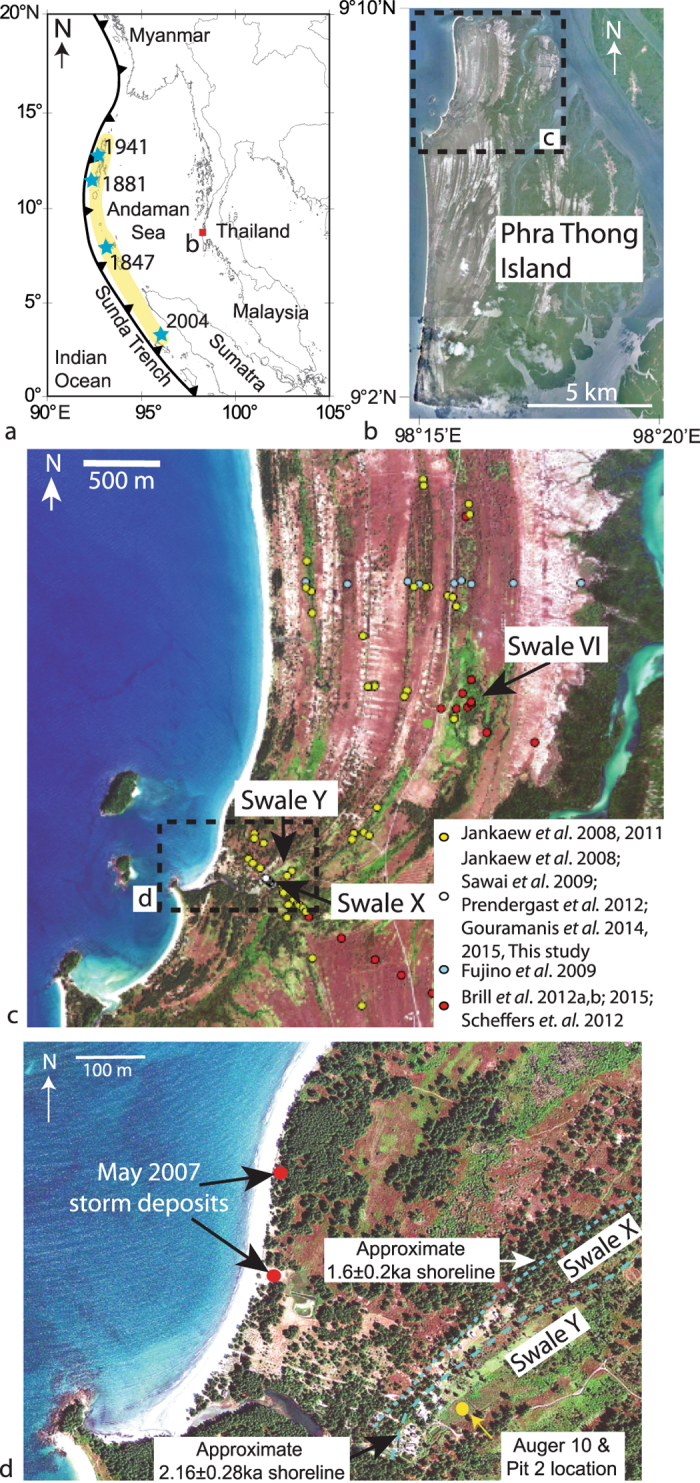
(**a**) Map (created in PanMap v0.96^59^) highlighting location of Phra Thong Island, the Sunda Trench and historical earthquake (1847, 1881, 1941 and 2004) epicentres^10^ (blue stars) and the 2004 Indian Ocean Tsunami slip patch (yellow shading), (**b**) Phra Thong Island, Thailand, showing the beach ridge sequence and highlighting the northern beach ridge sequence (satellite image courtesy of Google Earth Pro v. 7.1.5.1557, Image data: (C) 2016 DigitalGlobe, TerraMetrics, CNES/Astrium), (**c**) image of the northern beach ridge sequence and locations where previous work has been performed^20^,^21^,^27^,^28^,^29^,^30^,^32^,^36^,^39^,^44^,^45^ and the locations of Swales X, Y and VI (satellite image courtesy of GeoEye1, collected on the 7^th^ February 2012, GeoEye, Inc.), (**d**) detail of Swales X and Y and the location where Auger 10 and Pit 2 were excavated, locations of two 2007 storm fans (red dots) and approximate locations of previous (1.6 and 2.16 ka) shorelines from quartz OSL dates^36^ (dashed lines) (satellite image courtesy of GeoEye1, collected on the 7^th^ February 2012, GeoEye, Inc.).

**Figure 2:**
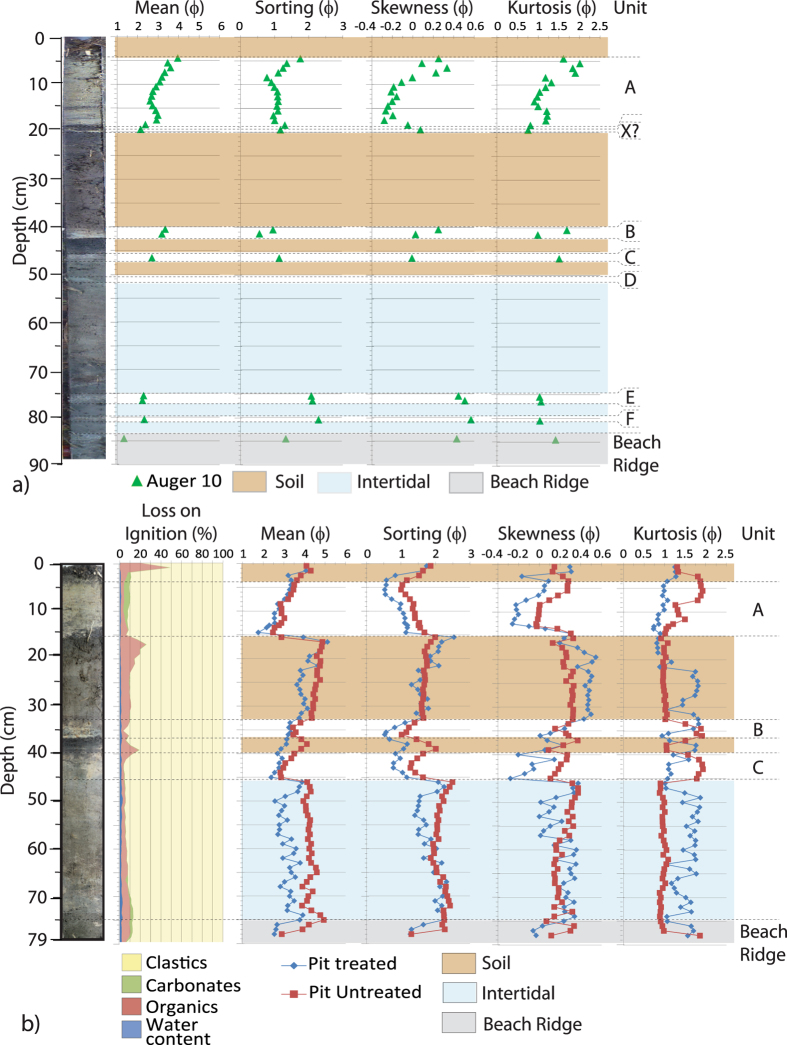
(**a**) Core photos, stratigraphy and sediment analysis of sand layers from Auger 10, (**b**) same for Pit 2.

**Figure 3:**
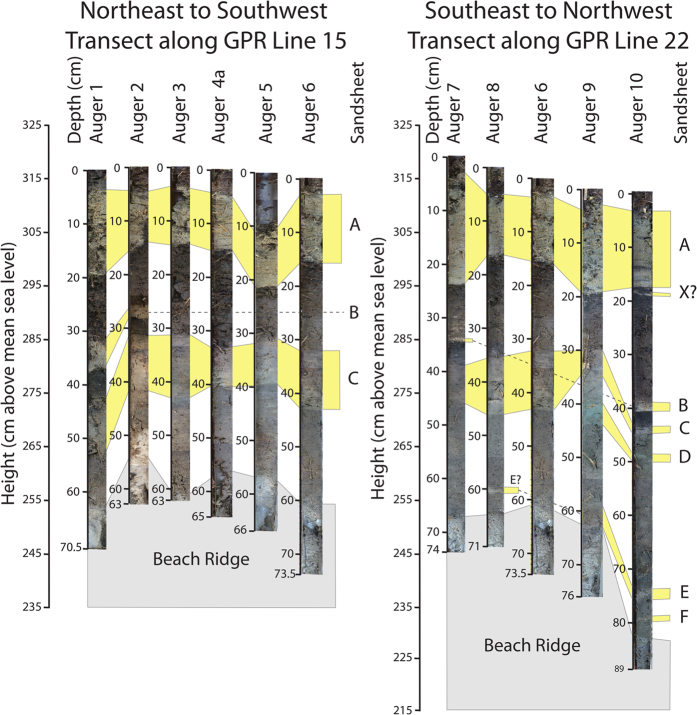
Stratigraphic correlation of the sand sheets recovered from the 10 auger cores collected^43^. Auger cores along two ground-penetrating radar (GPR) transects (Lines 15 and 22) were collected. Auger core 6 occurs at the intersection of the two GPR transects.

Sandsheet X was observed in pit KPT 37 from Swale VI^30^, and quartz OSL dated the layer as forming at 216 ± 23 years. This unit was interpreted as a lower subunit of the 2004 IOT sand sheet as it contained carbonate and unidentified fossils, and reworked plant material separated this sand layer from the overlying 2004 IOT sand sheet^30^. From Auger 10, a similar thin sand sheet has been recovered from an identical stratigraphic location between Sandsheets A and B (Figs 2a and 3), but this sand sheet was not observed in any other cores or dated. However, the sand sheet is constrained to have been deposited prior to 2004 but after *ca*. 1455 CE^17^. Although Sandsheet X is thin and only found in Auger 10, it is unlikely to have been deposited by a storm as the location of the swale between 1455 CE and 2004 was likely to have been between 100 to 200 m from the 2012 shoreline (Fig. 1d). No modern analogues exist to suggest such an expansive storm deposit on Phra Thong Island to have deposited sand in Swale Y. The sand sheet is unlikely to have been formed by a historical tsunami caused by an earthquake occurring in the northern segment of the 2004 IOT rupture zone (e.g. 1847, 1881 and 1941; Fig. 1)^10^,^30^,^46^. It was postulated that Sandsheet X was deposited during the 2004 IOT and that the organic layer formed from settling of material that was eroded by the initial incoming wave^30^. However, it is difficult to reconcile that the first wave of the 2004 IOT deposited such a small volume of clastic sediment and then reworked plant material, the source of which was not specified, before later waves emplaced the decimetre thick Sandsheet A unit above^30^. An alternative explanation for Sandsheet X in Swale VI and the sand sheet recovered from Auger 10 is that the sand sheet was formed by aeolian reworking allowing sands from the surrounding beach ridges to be blown into the swale. If the sole quartz OSL date from Sandsheet X of 216 ± 23 years is correct^30^, this places the formation of this sand sheet within the timeframes of the Strange Parallels Drought (*ca*. 1756–1758AD) and the East India Drought (1792–1796AD) as determined from tree-ring records^47^. From the tree ring record, the Strange Parallels Drought severely affected the west coast of Thailand, whereas the East India Drought was less severe^47^. Both droughts resulted in significant rainfall declines that would have reduced plant coverage of the beach ridges and allowed the remobilisation of sand into the swales.

The complex geophysical signature recorded by the high-frequency GPR analysis within Swale Y^44^ prompted a more detailed investigation into the deeper stratigraphy at this site from both Auger 10 and Pit 2 (Fig. 2).

The base of Swale Y has been variably dated using ^14^C analysed from three unabraided molluscan shells to give a date between 2500 to 2800 cal. yr. BP^21^, and a quartz OSL date of the basal sediment of 2.54 ± 0.34 ka that confirms the ^14^C dates^32^,^36^. The beach ridges on either side of Swale Y have also been quartz OSL dated with Ridge 3, i.e. the ridge landward of Swale Y, formed at 2.56 ± 0.35 ka and Ridge 2, i.e. the ridge seaward of Swale Y, formed at 2.16 ± 0.28 ka^36^ (Prendergast, A.L. pers. comm. The error in in the chronology in the Prendergast *et al*. 2012 paper is in the table. The ages shown in the figure are correct).

Of note, high-frequency GPR analysis has shown that the lee side of Ridge 2 has been scoured and sequentially rebuilt up to *ca*. 1 m after palaeo-tsunami inundation^44^,^45^. This was observed at locations where quartz OSL samples were collected between 0.4 and 0.6 m depth^36^. Also, there is extensive reworking of the sediments on the modern beach ridges by burrowing lizards and on the beach face by crabs, which may contribute to quartz OSL dating errors of beach ridges on Phra Thong Island^48^,^49^.

Sandsheet D has been quartz OSL dated at 2.1 ± 0.26 ka^36^ in Swale Y, and 1.85 ± 0.21 ka, 2.04 ± 0.28 ka and 1.8 ± 0.2 ka^30^ in Swale VI, suggesting that between the two swales the event was either diachronous, or if the sediments were deposited from the same event, the event occurred at *ca*. 2 ± 0.48 ka. Sandsheets E and F were deposited prior to Sandsheet D and after the formation of Swale Y, i.e. between 2.54 ± 0.34 ka and 2 ± 0.48 ka. If the outer errors are considered correct, then the postulated 550 to 700-year recurrence interval^21^ for great earthquakes originating from the Sunda Trench and producing 2004 IOT-type inundation and sedimentological deposits on Phra Thong Island cannot be discounted. If the mean ages or inner errors between the formation of Swale Y and the deposition of Sandsheet D are considered correct, then the potential recurrence interval generating Sandsheets E and F may be more frequent than the 550 to 700-year recurrence interval. If this is correct, then non-linear recurrence intervals for tsunami-generated washover deposits^42^,^50^,^51^ must occur for tsunami-type sediment deposition on Phra Thong Island. Further dating of Sandsheets E and F and surrounding sediments using current quartz OSL and ^14^C AMS dating techniques are unlikely to constrain the ages of these units.

However, the presence of the May 2007 storm washover fans extending between 20 and 50 m inland from the present day berm^35^ (Fig. 1) demonstrates that unusual storms can cause wave set-up to generate overwash and sediment deposition on Phra Thong Island. The age of Ridge 2 at *ca*. 2.16 ± 0.28 ka^36^ implies approximately 370 metres of beach ridges seaward of Swale Y have accreted at an assumed linear rate of 0.17 m/yr. If this rate is correct, then the beach face was within 30 m of the swale when Sandsheet D was deposited, and closer during the deposition of Sandsheets E and F. Mean sea level was also likely to have been less than 1 meter higher than modern sea level along the Malay-Thai coastline at *ca*. 2 ka^27^,^52^. The distance between the *ca*. 2 ka shoreline and the swale on the lee side of the immature beach ridge/berm crest is within the distance that recent storms have emplaced sediment along the modern shoreline. The back-beach environment was accumulating intertidal sands in this period and retained sufficient accommodation space to preserve thin overwash deposits. The distinct packages of clean sand comprising Sandsheets E and F, and the lack of sedimentary structures does not preclude the deposition of storm or tsunami as both causal mechanisms can deposit massive, sand-rich units in protected embayments^53^,^54^.

Due to the background intertidal sedimentation occurring prior to Sandsheet C deposition in Swale Y, other mechanisms of sand sheet emplacement cannot be discounted for the deposition of Sandsheets E and F. The modern intertidal channel that Swale Y drains towards is currently subjected to tidal variations and swash bar sedimentation. The close proximity of Swale Y to swash bar accretion and tidal reworking at the time of Sandsheets E and F emplacement cannot be discounted as a potential causal mechanism for the formation of these sand sheets. Similarly, the role of intense droughts^47^ resulting in sand reworked from the beach ridges also cannot be excluded. More recently, the role of meteotsunami has also been investigated^55^, but little work has been conducted to investigate this mechanism as potential cause of washover deposits. Each of these considerations remain at the forefront of palaeo-overwash investigation.

## Methods

Ten auger cores were collected in March 2013 from the south-western portion of Swale Y on Phra Thong Island, Thailand^44^. These augers variably penetrated 62 to 89 cm of the swale sediments (Fig. 3) and in each auger core, the basal, clean, coarse sands of the underlying beach ridge were collected, ensuring a complete stratigraphic sequence^44^. Several of the augers preserved sandsheets that could not be correlated with the known sandsheets from the previous work conducted on the island. Auger core 10 was selected for further sedimentological investigation as it contained the most sandsheets that were easily identifiable and very well preserved. At each augering location, detailed topographic data was collected using differential Global Position System (dGPS) with a maximum vertical error of 4 cm and maximum horizontal error of 1.5 cm.

Only sand layers preserved in the 89-cm long Auger 10^44^ (A10: 9°7′54.92″N, 98°15′44.44″E) were subsampled at 1-cm thick increments during March 2013. In May 2013, a second field trip was conducted to investigate the stratigraphy of the deeper sand layers observed in Auger 10. Two pits were dug (Pit 1: 9°07′54.850″N, 98°15′44.49″E; Pit 2: 9°07′54.93″N, 98°15′44.43″E), but, the groundwater table was higher in May and the base of Swale Y was not reached. A 78.5 cm sediment profile was collected from Pit 2 located less than 0.5 m northwest of Auger 10, and was photographed and subsampled at 1-centimetre increments. Sediments were not recovered from Pit 1.

Each sample from Pit 2 was analysed by loss on ignition (LOI), where crushed samples were sequentially heated to 105 °C (2 hours) to remove water, 550 °C (4 hours) to remove organics, and 950 °C (2 hours) to remove carbonate content^56^. After each heating, the samples were allowed to cool to room temperature before they were weighed and the percentage of water, organic and carbonate content were determined. The remaining sediment corresponds to the percentage of clastic material in each sample.

Each sample from the sand layers subsampled from Auger 10, and each centimetre from the Pit 2 profile were analysed for grainsize parameters. Initially, bulk sediment samples (Untreated) were analysed from Pit 2. Auger 10 samples and samples from Pit 2 were subjected to HCl treatment to remove carbonates, and several H_2_O_2_ treatments to remove organic material to determine the granulometry of the clastic component (Treated). This facilitates comparison between the clastic-only component of the sediment and the clastic + organic + carbonate components of the sediment (Supplementary Information). Grainsize was analysed on a Malvern Mastersizer2000 which uses laser obscuration to measure grainsize. Each sample was subjected to 1 minute sonication to further disaggregate the sediments. Each sediment analysis is replicated three times such that the relative standard deviation (RSD) of the mean grainsize (ϕ) between replicates is <1%, and the average of the three replicates is subsequently used. Where RSD is >1%, the sample is re-analysed until RSD achieves the <1% requirement. Each sediment analysis was analysed using GRADISTAT^57^ reported using the terminology of Folk and Ward^58^.

## Additional Information

**How to cite this article**: Gouramanis, C. *et al*. High-frequency Coastal Overwash Deposits from Phra Thong Island, Thailand. *Sci. Rep.* **7**, 43742; doi: 10.1038/srep43742 (2017).

**Publisher's note:** Springer Nature remains neutral with regard to jurisdictional claims in published maps and institutional affiliations.

## Supplementary Material

Supplementary Information
