# Supplementary material for: High-frequency Coastal Overwash Deposits from Phra Thong Island, Thailand
Source: Sci Rep. 2017 Mar 9;7:43742. doi: 10.1038/srep43742 (PMC5343490; doi:10.1038/srep43742)
Supplement: Supplementary Information [file srep43742-s1.pdf]

Supplementary Information

High-frequency Coastal Overwash Deposits from Phra Thong Island, Thailand

Chris Gouramanis[1*], Adam D. Switzer[2,3], Kruawun Jankaew[4], Charles S. Bristow[5], Pham T. Dat[2,3] and Sorvigenaleon R. Ildefonso[2]

1. Department of Geography, National University of Singapore, Singapore, 117570

2. Earth Observatory of Singapore Nanyang Technological University Singapore, Singapore, 639798

3. Asian School of the Environment Nanyang Technological University Singapore, Singapore, 639798

4. Department of Geology Chulalongkorn University Bangkok, Thailand, 10330

5. Department of Earth and Planetary Sciences Birkbeck College, University of London London, United Kingdom, WC1E 7HX

* Corresponding author, geogc@nus.edu.sg

Auger 10 Stratigraphy

The stratigraphy of the lower section of Auger 10 differs from both of the general stratigraphy presented by Jankaew *et al*. (2008) and that recorded in Pit 2. We include descriptions of the d_{10} and d_{90} sediment fractions here as this information corroborates the granulometric properties discussed in the text (Supp Info Fig. 1a).

Auger 10 penetrated the base of the swale at 84 cm and the basal sediments are clean, medium grey, poorly sorted, very finely skewed, leptokurtic, quartz-rich, medium sands reflecting the underlying beach ridge sediments (Gouramanis *et al*. 2015). From 84 to 80.5 and 80 to 77 cm, the sediments consist of quartz-rich, dark grey, silty fine sand, with small black charcoal or diagenetically altered plant material, and are similar to the intertidal sediments described from Pit 2.

Two thin, light grey, clean, quartz-rich, very poorly sorted, very finely skewed, mesokurtic fine to medium sand layers occur from 80.5 to 80 cm and 77 to 75 cm depth. The d_{10} and d_{90} fractions of these sediment samples are slightly coarse tailed and are very similar to the underlying beach-ridge sediments, suggesting a local provenance. The lower contact of each layer is sharp, but the upper contact grades into the overlying sediments. The layers are named Sandsheets F and E, respectively.

Above Sandsheet E, extending to 68 cm depth is a dark grey silty very fine sand dominated by clastic minerals and plant fibres. Between 72 and 71 cm, the layer contains a ovoid, dark brown silty very fine sand structure aligned such that the observed long axis is horizontal to the bedding and may be a relict burrow.

From 68 to 51 cm and 50 to 46 cm depth is a medium grey-brown silty fine sand dominated by clastic minerals and speckled with small black charcoal or diagenetically altered plant material throughout. These sediments are similar to the unit described between 79 and 45 cm in Pit 2 and the intertidal sands of Jankaew *et al*. (2008).

Between 51 and 50 cm is an inclined, gun-metal blue, quartz-rich, silty fine sand with sharp upper and lower contacts. This sandsheet is found in other auger cores collected from Swale Y (A8 and A9 located less than 1.5 metres to the southeast of A10; and PTHA4 and PTHA5 collected from approximately 15 m to the northeast of A10; Gouramanis *et al*. 2015). The distinctive colouration and stratigraphic positioning, wholly contained within the intertidal sands, suggests that this sand sheet is a correlative of Jankaew *et al.*'s (2008) Sandsheet D found in Swale Y.

Between 46 and 45 cm, is a thin layer of clean, light grey, quartz-rich, poorly sorted, symmetrically distributed, very leptokurtic fine sand. The d_{10} and d_{90} component suggest a subtle skewing of the sediments towards a slightly coarser grained tail. Both of the basal and upper contacts are sharp. This layer corresponds to Sandsheet C in Pit 2, other nearby auger cores (Gouramanis *et al*. 2015) and from Jankaew *et al*.'s (2008) study. Above this sand layer is a 4.5 cm thick, dark brown organic-rich, muddy fine sand layer.

At 41.5 cm, a sharp contact exists between the organic muddy sands below and a two-centimetre thick, clean, white, moderately sorted, finely skewed to symmetrical, and very leptokurtic to mesokurtic grainsize distribution, very fine sand. The d_{10} and d_{90} fractions attest to the symmetric distribution of the sediments. This sand layer corresponds to Sandsheet B (Jankaew *et al*. 2008).

Sandsheet B grades into the overlying dark grey-brown organic-rich silty sands, that extends from 39.5 to 19.5 cm and from 19 to 18 cm depth. The high content of plant stems, leaves and rootlets indicates the continued deposition or organic material within the protected swale.

Between 19.5 and 19 cm is a clear, tan, poorly sorted, symmetrical and platykurtic distribution of fine sand, that is similar to Brill *et al.*'s (2012-NHESS) Sandsheet X. This unit also displays a coarser d_{10} and d_{90} sediment grain size component indicating a coarser-tailed grain size distribution than the intervening organic muds.

From 18 to 4 cm is a series of five white layers of quartz-dominated moderately to poorly sorted, coarse to very finely skewed, leptokurtic to platykurtic fine sand grading upwards to very fine sand. Very thin bands of dark organic-rich material occur at 16.5, 15 to 14 and 12.5 cm. This unit corresponds to Jankaew *et al.*'s (2008) Sandsheet A deposited during the 2004 IOT. This layer also contains abundant plant debris. The d_{10} fraction of this unit fines upward and the d_{90} fraction coarsens to 13.5cm, then fines to 6.5 cm before slightly coarsening to 4 cm. Thus, the lower sediments in the 2004 tsunami deposit have entrained a coarser fraction of the sediments and these lower sediments have a coarser-tailed distribution.

Above this sand sheet is a dark brown organic layer containing modern leaves, stems and roots of modern plants, representing the accumulation of organic matter after the 2004 IOT and providing a modern analogue for the lower organic units.

Pit 2 Stratigraphy

The stratigraphic lowest unit in Pit 2 is a pale to medium grey clastic (2 to 9% organic matter, <3% carbonate), poorly to very poorly sorted, very finely skewed to symmetrically distributed, very leptokurtic to mesokurtic medium silty to very coarse silty fine sand that extends from 79 to 45 cm depth and has been interpreted as in intertidal deposit (e.g. Jankaew *et al.* 2008).

From 45 to 40 cm, the sediment is a tan, quartz-rich (<3% organics, <1% carbonate), poorly to moderately sorted, very fine to coarsely skewed, mesokurtic to leptokurtic coarse silt to fine sand corresponding to Sandsheet C (Jankaew *et al*. 2008).

From 40 to 37 cm, a dark brown, organic-rich (8.5 to 17% organic material, <1.5% carbonate), moderately well sorted, leptokurtic fine to very fine sandy unit occurs that is interpreted as organic mud accumulation under freshwater, swampy conditions that prevailed in the swale between deposits of sand sheets (Jankaew *et al*. 2008).

Between 37 and 34 cm is a tan with dark brown mottled quartz-rich unit (<7% organics, <1% carbonate), moderately sorted, symmetrical to finely skewed, very leptokurtic to mesokurtic, very fine sand interpreted to represent Sandsheet B, the penultimate palaeotsunami deposit (Jankaew *et al*. 2008).

The basal contact of this sand layer is sharp, but the upper contact grades into the overlying silty sand. From 34 to 22 cm is a grey-brown, poorly sorted, very finely skewed, mesokurtic to very leptokurtic, coarse silty very fine sand composed predominantly of clastics (6 to 9.5% organics, <1% carbonates). This unit may be heavily reworked Sandsheet B. The upper contact of this unit is inclined with the overlying dark brown organic-rich layer.

From 22 to 16 cm is a dark brown, organic-rich (11 to 25% organics, <1% carbonate), poorly to very poorly sorted, fine to very finely skewed, playkurtic muddy sand to sandy mud, signifying a return to typical swale-like organic mud accummulation (Jankaew *et al*. 2008).

From 16 to 4 cm depth, a tan, quartz-rich (<7.5% organics, <6% carbonates), poorly to moderately well sorted, symmetrical to coarsely skewed, platykurtic to mesokurtic medium to fine sand corresponds to the 2004 IOT deposit, Sandsheet A. This deposit has a sharp, inclined basal contact, and from the base to the top of the deposit sorting improves, grain distributions trend to better symmetry and the sediments fine upwards.

The upper 4 cm contains a reworked moderately to poorly sorted, very finely to coarse skewed mesokurtic and leptokurtic muddy sands increasing in organic content (<7.5% below 2 cm and <48% in the upper 2 cm) organics and <5.5% carbonates. This unit represents the organic accumulation that has occurred within the swale since 2004 (Jankaew *et al*. 2008). Modern roots and plant fibres permeate the stratigraphy.

The d_{10} and d_{90} fractions of the overwash sediments preserved in Pit 2 match the d_{10} and d_{90} fractions of the overwash sediments of Auger 10 (Supp Info. Fig. 1b). The grain size distributions of each of the organic samples above 40 cm depth are symmetric with very fine d_{10} fraction and fine d_{90} fraction. The intertidal sediments below Sandsheet C (45 cm depth) are coarse tailed, but less so than the lower overwash deposits or the base of the 2004 IOT deposit.

Treated versus Untreated Samples

The analysis of treated and untreated sediments recovered from Pit 2 (Fig. 2b) and the comparison of the granulometric parameters (Supp. Info. Fig. 2) demonstrates some important features that can confound the interpretation of tsunami and palaeotsunami deposits and the background sediments.

Comparing the different granulometric parameters from Pit 2 indicates that certain comparisons, i.e. Mean versus Sorting (Supp. Info. Fig. 2a), Mean versus Kurtosis (Supp. Info. Fig. 2c), Sorting versus Kurtosis (Supp. Info. Fig. 2e) and Skewness versus Kurtosis (Supp. Info. Fig. 2f), would distinguish two sediment populations in the Untreated analysis. However, with the removal of carbonates and organics after chemical treatment, the distinct sediment populations disappear (Supp. Info. Figs. 2a-f).

As mean/median grain size and sorting are commonly used to discriminate between tsunami/palaeotsunami and storm and background sediments (e.g. Nanayama *et al*. 2000; Goff *et al*. 2004; Switzer *et al*. 2005; 2008; Szczucinski *et al*. 2012), with background sediments being more poorly sorted (e.g. Switzer *et al*. 2005), or better sorted than the overwash deposits (e.g. Nanayama *et al.* 2000; Switzer *et al.* 2008), or inferred tsunami deposits having poorer sorting than storm deposits (Goff *et al*. 2004).Of note, using HCl and H_2O_2-treated sediment samples sorting and median grainsize could not discriminate the background 'soil' units from the sediments deposited by the tsunami on the Sendai Plain, Japan, after the 2011 Tohoku-oki tsunami (Szczucinski *et al*. 2012).

From Pit 2 on Phra Thong Island (Fig. 2b), individual units show negligible internal variability in each of the granulometric parameters in the untreated (red) analyses and greater internal variability in the treated (blue) analyses. Both Sandsheet A and Sandsheet C are apparent in both treated and untreated samples with major excursion of mean grain size, sorting and skewness, whereas the kurtosis only shows a pronounced excursion only in the untreated analyses.

Although clearly evident in the sediment photos (Fig. 2b), Sandsheet B does not show a marked excursion in mean grain size in the treated samples but a distinct coarsening is apparent in the untreated analyses. The skewness analyses of Sandsheet B show an anti-phased relationship, with skewness sequentially increasing in the treated samples and decreasing in the untreated samples. Sandsheet B may be thicker than is observed in the photos with the upper 12 cm (34 to 22 cm) being heavily reworked and mixed with a higher proportion of organics (*ca*. 10%). If this is the case, then this mixed zone is only observed in the treated analyses and is masked by the untreated granulometric analyses.

The kurtosis of each sample analysed from Pit 2 (Fig. 2b) records an antiphased relationship between treated and untreated samples. The kurtosis is low in Sandsheets A, B and C and increases in the background sedimentological units in the treated samples, but shows an opposite relationship when the samples are untreated.

Each of the granulometric analyses of the treated and untreated sediment samples from Pit 2 on Phra Thong Island, highlight the utility of using granulometry of bulk and clastic sediments to extract important information on the sedimentology and stratigraphy of the overwash deposits. However, the comparison of granulometric parameters can introduce artefacts into the data if only untreated samples are examined (Supp. Info. Figs. 2a-f).

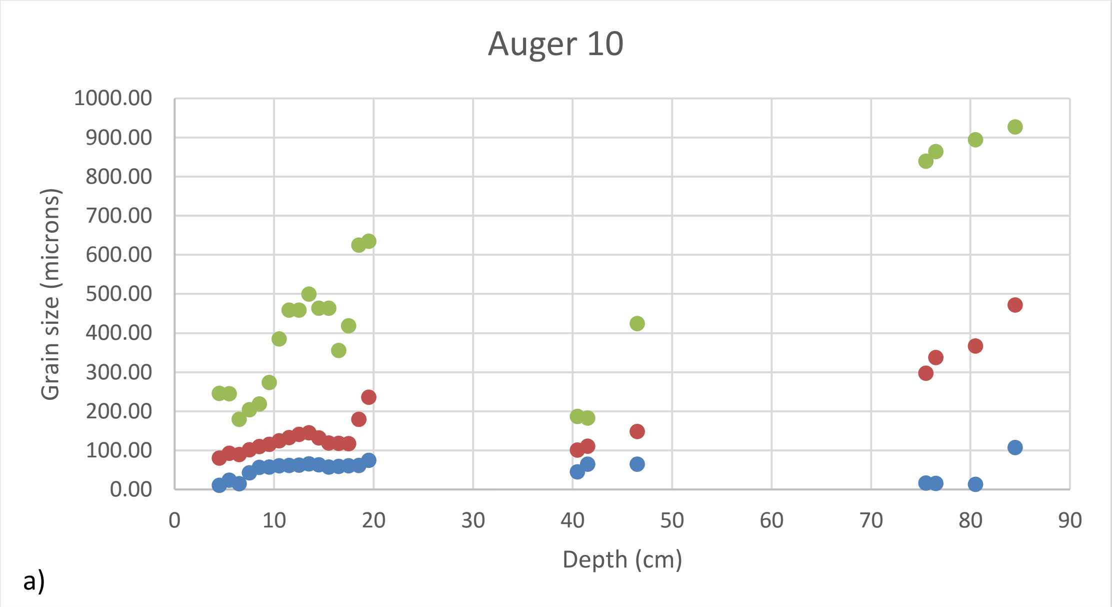

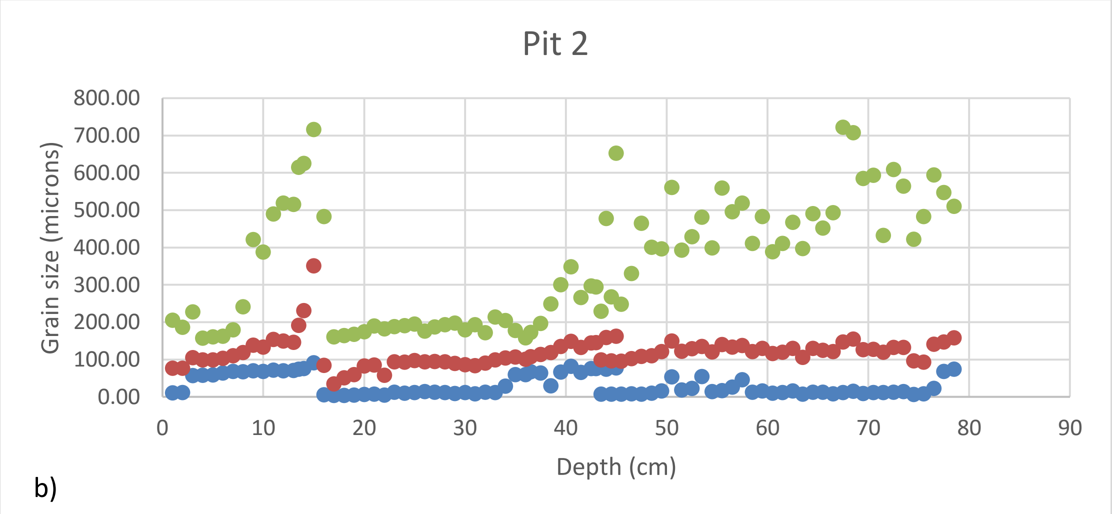

Supp. Info. Fig. 1. Grain size fractions d_{10} (blue dots), d_{50} (red dots) and d_{90} (green dots) for a) Auger 10 and b) Pit 2 sedimentological analyses.

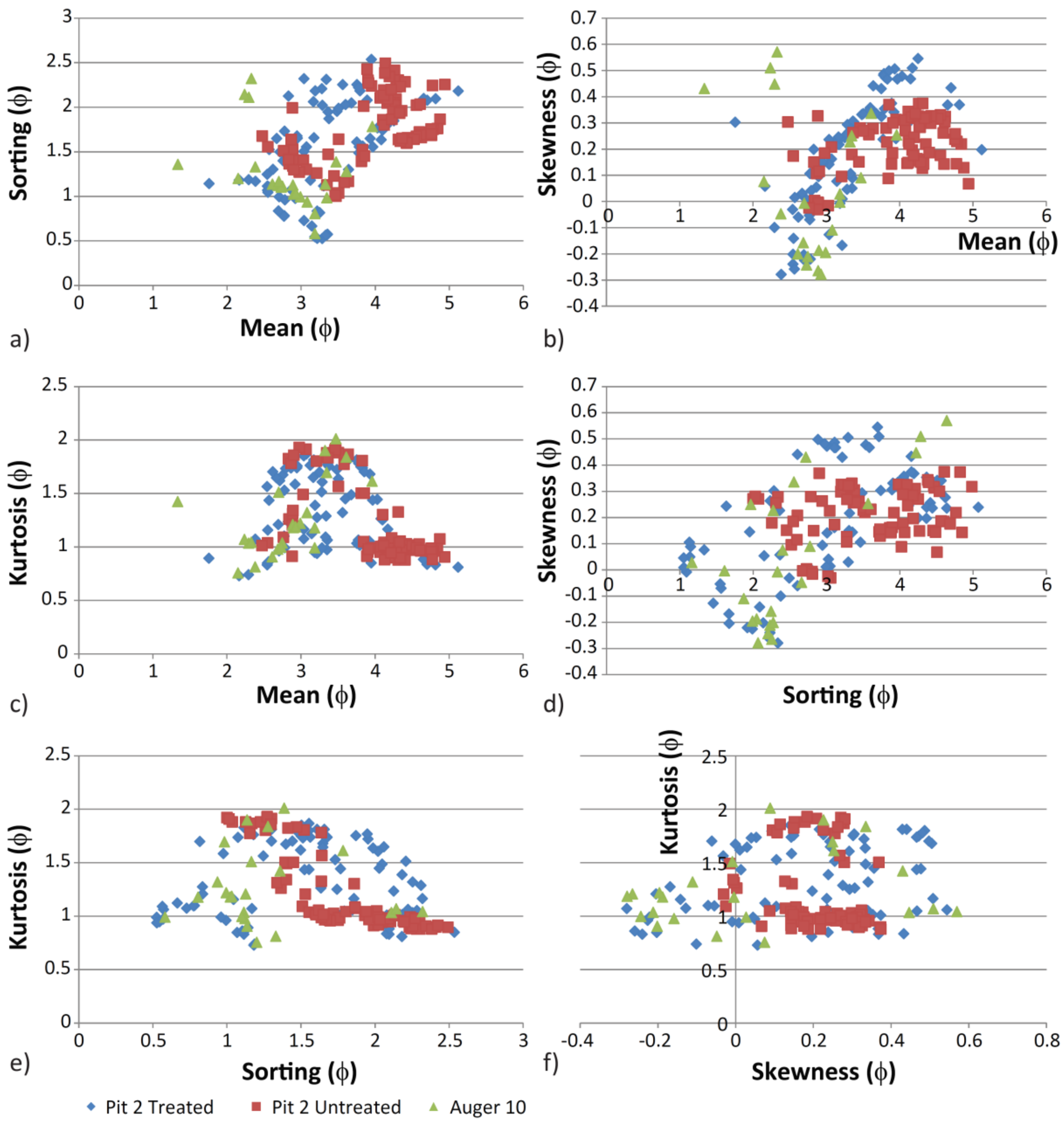

Supp. Info. Fig. 2. Comparison of the granulometric parameters measured from treated (blue diamonds) and untreated (red squares) from Pit 2 and treated samples from the sand layers in Auger 10, a) Mean grainsize versus Sorting, b) Mean grain size versus Skewness, c) Mean grain size versus Kurtosis, d) Sorting versus Skewness, e) Sorting versus Kurtosis, and f) Skewness versus Kurtosis.

References

Brill, D., Klasen, N., Jankaew, K., Brückner, H., Kelletat, D., Scheffers, A., Scheffers, S., 2012b. Local inundation distances and regional tsunami recurrence in the Indian Ocean inferred from luminescence dating of sandy deposits in Thailand. Natural Hazards and Earth System Science 12, 2177-2192.

Goff, J., McFadgen, B.G., Chagué-Goff, C., 2004. Sedimentary differences between the 2002 Easter storm and the 15th-century Okoropunga tsunami, southeastern North Island, New Zealand. Marine Geology 204, 235-250.

Gouramanis, C., Switzer, A.D., Polivka, P., Bristow, C.S, Jankaew, K., Pham, T.D. Lee, Y.S., Rubin, C. & Ramos, I.S. (2015). Ground Penetrating Radar examination of thin tsunami beds – A case study from Phra Thong Island, Thailand. Sedimentary Geology 329: 149-165.

Jankaew, K., Atwater, B.F., Sawai, Y., Choowong, M., Charoentitirat, T., Martin, M.E., Prendergast, A., 2008. Medieval forewarning of the 2004 Indian Ocean tsunami in Thailand. Nature 455, 1228-1231.

Nanayama, F., Shigeno, K., Satake, K., Shimokawa, K., Koitabashi, S., Miyasaka, S., Ishii, M., 2000. Sedimentary differences between the 1993 Hokkaido-nansei-oki tsunami and the 1959 Miyakojima typhoon at Taisei southwestern Hokkaido northern Japan. Sedimentary Geology 135, 255-264.

Switzer, A.D., Pucillo, K., Haredy, R.A., Jones, B.G., Bryant, E.A., 2005. Sea Level, Storm, or Tsunami: Enigmatic Sand Sheet Deposits in a Sheltered Coastal Embayment from Southeastern New South Wales, Australia. Journal of Coastal Research 214, 655-663.

Switzer, A.D., Jones, B.G., 2008. Large-scale washover sedimentation in a freshwater lagoon from the southeast Australian coast: sea-level change, tsunami or exceptionally large storm? The Holocene 18, 787-803.

Szczuciński, W., Kokociński, M., Rzeszewski, M., Chagué-Goff, C., Cachão, M., Goto, K., Sugawara, D., 2012. Sediment sources and sedimentation processes of 2011 Tohoku-oki tsunami deposits on the Sendai Plain, Japan — Insights from diatoms, nannoliths and grain size distribution. Sedimentary Geology 282, 40-56.
